# Genomic Assays in Node Positive Breast Cancer Patients: A Review

**DOI:** 10.3389/fonc.2020.609100

**Published:** 2021-02-16

**Authors:** Maroun Bou Zerdan, Maryam Ibrahim, Clara El Nakib, Rayan Hajjar, Hazem I. Assi

**Affiliations:** ^1^ Department of Internal Medicine, Naef K. Basile Cancer Institute, American University of Beirut Medical Center, Beirut, Lebanon; ^2^ Division of Internal Medicine, Massachusetts General Hospital and Harvard Medical School, Boston, MA, United States

**Keywords:** breast cancer, oncotype DX, MammaPrint, PAM50, Endopredict, breast cancer index, genomic grade index, immunohistochemistry

## Abstract

In recent years, developments in breast cancer have allowed yet another realization of individualized medicine in the field of oncology. One of these advances is genomic assays, which are considered elements of standard clinical practice in the management of breast cancer. These assays are widely used today not only to measure recurrence risk in breast cancer patients at an early stage but also to tailor treatment as well and minimize avoidable treatment side effects. At present, genomic tests are applied extensively in node negative disease. In this article, we review the use of these tests in node positive disease, explore their ramifications on neoadjuvant chemotherapy decisions, highlight sufficiently powered recent studies emphasizing their use and review the most recent guidelines.

## Introduction

We have come a long way in the emerging genomic paradigm! We are no longer at crossroads in the management of malignancies, especially breast cancer (BCA). Previously, we solely referred to the combination of the patient’s clinical presentation along with her pathological findings when treating BCA patients. Today, we are leaping a huge step towards integrating genomic assays in node -ve patients’ management. In this review, we further explore the potential role of multiple genomic assays in node positive disease as well.

BCA remains the prominent disease among females globally, epitomizing about 25% of all malignancies (1). Incidence rates (IR) differ across different countries. While the IR is 0.025% in Central Africa and East Asia and 0.092% in Western Europe, the age-standardized IR of BCA is 45.3 per 100,000 females in the region and is considerably rising, with estimates to touch Western numbers (2, 3). Incident cases are projected to rise globally by at least 46.5% in the next 20 years (4). With the progresses in BCA screening techniques, cancer detection rate has improved. This has led to a decrease in mortality from BCA due to both earlier detection and systemic individualized treatments (5). Deciding when to go for endocrine therapy (ET), adjuvant chemotherapy (CT), surgery or combining them can be challenging. In general, most BCAs ≤0.5 cm in size have a better prognosis with hormonal therapy alone and do not characteristically necessitate adjuvant chemotherapeutic treatment (5, 6). At one end of the spectrum, all estrogen receptor (ER) +ve, progesterone receptor (PR) +ve, HER2 -ve, node -ve BCAs <1 cm in size are treated similarly (7). At the other end, most patients with stage III BCAs will necessitate adjuvant chemotherapeutic treatment because of their recurrence risk and the likely benefits of chemotherapeutic management (6). The majority of cases of ER +ve BCA fall in between these two extremes and thus warrant gene expression profiles for further decision-making (8). Whether neoadjuvant CT should be administered or not remains a question without a complete answer, but ongoing research is bridging us closer to an individualized approach for every patient.

Before discussing the different biomarker assays used to lead conclusions on adjuvant systemic treatment related to females with early invasive disease, it’s imperative to differentiate between prognostic and predictive biomarkers (9, 10). The former reflects disease recurrence or progression independent of any therapy received (10, 11). On the other hand, a biomarker is predictive if therapy leads to an incongruent outcome in patients that are biomarker +ve in comparison to patients that are biomarker -ve (10, 11). We limit our discussion to the most commonly used genomic assays.

## Discussion

### Oncotype DX

#### General Information

Oncotype DX (Genomic Health, Redwood, CA) is one of the widely applied multiplex BCA testing tools. It was initially put on a trial basis in 2007 and became widely available in 2011. Today, it is incorporated in several staging, diagnosis and/or treatment guidelines for early BCA, including guideline from the American Joint Committee on Cancer (AJCC) 8th edition, American Society of Clinical Oncologists (ASCO), National Comprehensive Clinical Network (NCCN), European Society of Medical Oncologists (ESMO), National Institute for Health and Care Excellence (NICE), and St Gallen’s (12). It was developed by first identifying the 250 most promising candidate genes out of 447 tumor samples from patients from three separate studies (13). These genes were then limited down to 21 genes split into two sets. The first set was comprised of 16 cancer related genes, which were also subdivided into five subgroups. The subgroups include proliferation genes (Ki67, STK15; Survivin, CCNB1, MYBL2), invasion genes (MMPP11, CTSL2), HER2 genes (GRB2, HER2), estrogen genes (ER, PGR, BCL2, SCUBE2), and other cancer related genes (GSTM1, CD68, BAG1). The second group covers reference genes: ACTB, GAPDH, RPLPO, GUS, and TFRC. It is based on RNA isolation from Formalin-Fixed Paraffin-Embedded (FFPE) BCA tissue followed by RT-PCR. It derives a recurrence score (RS) which optimizes prediction of distant relapse despite tamoxifen therapy (8). Based on RS, ER +ve/HER2 -ve BCA, with or without limited nodal involvement, patients are recommended advised to proceed with ET for a period of ≤5 years (5). To note, Oncotype DX has been readily used to classify cases of tumors ≤5 cm with no metastasis with an RS <11 as a pathological stage IA (14). For women older than 50 years with an RS of 0–25 or women less than 50 years old with an RS of 0–15, it is probable that the benefits of CT will not offset the risks of adverse effects, and that the disease has a low risk of relapse (15). For women older than 50 years with an RS of 26–100 or women less than 50 years with an RS of 26–100, it is probable that the benefits of CT will compensate the risks of adverse effects, and that BCA has a high risk of recurrence (RoR) (8). The approach to patients aged ≤50 years with node -ve cancers with RS between 16 and 25 remains controversial. To note, risk scores cut-offs have been adjusted in recent clinical trials, predicting an impending adjustment in the assay.

#### Validation Studies

In a study involving 2,892 patients having ER +ve lymph node (LN) –ve BCA, Paik et al. revealed if adjuvant tamoxifen provided a survival benefit in patients when followed up for 10 years (16, 17). RT-PCR was done on 668/675 FFPE tumor samples taken from patients receiving tamoxifen in the National Surgical Adjuvant Breast and Bowel Project B-14 randomized trial (NSABP-14) (16, 17). To note this was the first validation study related to this assay’s prognostic element (17). Approximately half of the patients (51%) were identified as having low-risk disease based on RS having a 10-year distant-recurrence rate (DRR) of 6.8% (16). The rest of the patients were stratified into intermediate-risk (22%) and high-risk (27%) having a 10-year DRR of 14.3 and 30.5% respectively. The absence of benefit from added therapy was independent of age or tumor characteristics; RS was an independent predictor of overall survival (OS) (P < 0.001) (17, 18). The importance of focusing on a HR +ve patient population when utilizing Oncotype DX was done by Esteva et al. in 2005 (19). Since this study involved patients having HR+ve and HR-ve BCA, it was the only study that failed to provide prognostic value of RS in patients with LN –ve disease not receiving adjuvant therapy. Last, in another study done by Paik et al. Oncotype DX was utilized on FFPE tumor samples from 651 patients out of 2,306 ER +ve and LN –ve BCA patients enlisted in the NSABP-20 trial, the first validation study for this assay’s the predictive component (17). Patients receiving CT with a high RS (≥ 31) had a 27.6% decrease in absolute risk along with a significant reduction in risk of distant recurrence (DR) at 10 years; relative risk (RR) 0.26; 95% CI 0.13–0.53 (16, 20). Inconclusive results were derived concerning patients with an intermediate RS (18–31), and patients with low RS (< 18) had an increase in absolute risk of 1.1% (20).

#### Role in Node Positive Disease

When it comes to patients with +ve LNs, some studies have shown encouraging results as well. A prospectively designed translational study on samples from the ATAC trial, known as the transATAC study, involving postmenopausal women with invasive BCA first comes to mind (21). This study evaluated 1,231 tumor samples out of 9,366 patients enrolled in the main ATAC trial (22). Of those samples, 243 patients had one to three +ve LNs and 63 patients had four or more +ve LNs. There was a statistically significant difference in risk of DR in these patients (14). The 9-year risk of DR was 17, 28, and 49% for patients with an RS strictly less than 18, RS between 18 and 30, and RS equal to or greater than 31, respectively (14). Both patients with node -ve disease and patients with 1–3 +ve LN along with low RS had estimates of 9-year risk of DR. Patients with ≥4 +ve LNs had a higher risk of DR for any RS result (14). What is more, non-dependent data was provided by RS result on the risk of DR in both two monotherapy arms of ATAC (22, 23).

SWOG 8814 trial was another trial to display encouraging results concerning the use of Oncotype DX with node +ve disease (24). Females aged 51 and above with axillary LN +ve, ER +ve disease were enrolled in this randomized clinical trial (25). It was one of the first prospective studies revealing the predictive evidence of Oncotype Dx for the benefit of CT in patients treated with ET (26). This study also recommended CT for patients with a high RS score more than 31, and it showed that no benefit of CT in patients with RS less than 11. To conclude, no optimal cut-off for patients having an RS between 11–25 was found concerning CT administration (27).

Wang et al. also conducted a retrospective study on LN +ve population using the Surveillance, Epidemiology, and End Results (SEER) 18 database. After identifying more than 4,000 patients diagnosed over 8 years from 2004 to 2012 with a median follow up of 57 months and using RS cut-offs from the TAILORx trial, a positive correlation was found between the RS risk groups and the pathologic prognostic stage (14, 28). Unlike low-risk RS patients, differences were found in Breast Cancer Specific Survival (BCSS) and OS in patients with intermediate-risk RS. As for patients with a pathologic prognostic stage IA, significant differences were found in BCSS and OS by RS (14, 28). These differences are most likely due to the high RS patients having inferior survival, supporting the use of RS to modify the pathologic prognostic stage irrespective of LN status (14, 28).

On the other hand, different RS ranges were used in the Trial Assigning Individualized Options for Treatment [Rx] (TAILORx) trial, and it interestingly revealed that the 9-year BCSS was >97% irrespective of LN status. The intermediate RS range shifted from 18–30 to 11–25 reducing the prospective chance of undertreatment; a 10% recurrence risk was represented by an upper confidence limit for RS 11 (29). More than 80,000 patients were treated over a 10 year period and followed up for a median of 49 months (14). This trial further emphasized tumor biology, especially for early-stage disease, over anatomic spread of the tumor and also calls for altering the pathologic prognostic stage using RS in LN +ve patients (14). Here, it is worth noting that Oncotype DX RS has been validated for predicting the advantage of complementing adjuvant CT to further decrease the RoR in patients with an RS 11–25 which makes it both predictive and prognostic (30–33). This was based on the TAILORx trial which showed an OS of 93.9 and 93.8% at 9 years with ET and chemoendocrine therapy, respectively (33). Similarly, there was also no statistically significant difference between ET or chemoendocrine therapy at 9 years for freedom from DR (94.5 and 95.0%) or at a distant or local–regional site (92.2 and 92.9%) (33). When further segregated into subgroups, patients ≤50 years of age having an RS of 16–20 or 21–25 were found to have a superior invasive disease-free survival (IDFS) by 2.7 and 5.8% (p = 0.004) respectively (33). Here it is worth noting that these findings could be explained by ovarian suppression caused by CT as previously found in the SOFT/TEXT trials (34). What is more, these findings have added to the intricacy of taking Oncotype DX’s recommendation in the mentioned age group (29, 33). Sparano et al. further stratified patients ≤50 years of age from the TAILORx trial, which provided level 1 data, with similar RS scores according to clinical risk (CR) (29, 35). It was found that clinically low-risk patients with RS 16–20 would not benefit from CT at 9 years while clinically high-risk patients having RS 16–20 would do so similar to patient having RS 21–25 (35). Panellists from the Breast Cancer Therapy Expert Group (BCTEG) realize that even though the standard of care is to administer CT to patients having an RS of 26–30, no prospective data exists supporting this recommendation (29).

In the West German Study Group (WSG) Plan B prospective trial, 110 patients with hormone receptor (HR) +ve, Her2 -ve, LN +ve tumors and an RS ≤11 showed a 5-year disease-free survival of 94.4% and a 3-year disease-free survival of 98% when treated with hormonal monotherapy (36, 37). Here it is worth mentioning the WSG- ADAPT trial: Adjuvant Dynamic marker-Adjusted Personalized Therapy trial Optimizing Risk Assessment and Therapy Response Prediction in Early Breast Cancer (38). This trial recruited approximately 4,900 patients using an innovating “umbrella” protocol design. Patients were recruited into three different sub-trials based on their diagnostic core biopsy: HR+/Her2-ve group, HR+/Her2+ve group and HR-/Her2+ve group (38). The first efficacy results were reported at the recent 2020 San Antonio Breast Cancer Symposium. After 3 weeks of pre-operative ET, patients with clinically high-risk LN-ve or LN+ve (≤ 3) BCA received ET only if RS was 0–11 or RS was 12–25 along with a Ki67 response. The 5-year distant disease-free survival was 96% irrespective of age.

Another trial, the RxPONDER trial, SWOG S1007 trial, randomized patients with 1–3 +ve nodes along with RS ≤25 to either receive CT or not along with hormonal therapy (14, 39). Results are highly anticipated, and some centres have already used the study’s inclusion criteria to omit patients from receiving CT even though the trial’s publication results are not out yet (40). During the last San Antonio Breast Cancer Symposium held in 2020, the latest update form the RxPonder trial was presented. This study evaluated patients who are at a higher absolute risk compared to the TAILORx trial, which assessed patients with a lower risk. Postmenopausal BCA patients with 0–3 +ve LNs having a low RS (≤ 25) did not benefit from adjuvant CT in any subgroup (41). The 5-year IDFS rate was 91.6 and 91.9% for postmenopausal patients on chemo-endocrine therapy vs ET only respectively (41). On the other hand, premenopausal BCA patients having a similar RS had a benefit from adding CT to ET. For premenopausal women, there was a significant benefit from CT in IDFS regardless of RS (41). The patients receiving chemo-endocrine therapy had a 5-year IDFS of 94.2 vs 89% in the ET group. Observed across premenopausal subgroups, the decrease in IDFS events was 46%. In addition, a 53% decrease in mortality was observed accounting to a 5-year OS absolute improvement of 1.3% (41). In other words, the 5-year OS rate was 98.6 vs 97.3% in the chemo-endocrine group vs the endocrine group, respectively. With that being said, in postmenopausal patients, CT is recommended for patients having an RS of 26–100 and ET is recommended for the rest of the patients RS ≤25. In premenopausal patients with an RS of 26–100, CT is recommended (41). In premenopausal patients with an RS less than 26 and +ve LNs CT is recommended as long as overall free survival is discussed; aromatase inhibitor can be considered as an alternative (41). As for premenopausal patients with LN –ve disease, patients with low CR and RS ≤21 or high CR and RS ≤16 are suggested to receive ET. As for patients with node -ve disease, low CR and RS 21–26 or high CR and RS 16–26, CT is recommended as long as overall free survival is discussed; aromatase inhibitor can be considered as an alternative (41). These findings take us closer to our aim of having targeted treatment and dropping the one-size-fits-all treatment approach. Even though these are still preliminary findings, the results are heavily anticipated. There are already a few propositions trying to explain these findings. Could the absolute improvement in OS observed in premenopausal women have resulted from the suppression of ovarian function by CT?

Here it is worth mentioning that according to ASCO, Oncotype DX should not be applied in patients who have LN +ve BCA (42). NCCN guidelines concerning Oncotype DX are the following: for patients with an intermediate or high RS, i.e., RS of 18–30 or RS ≥31, adjuvant CT should be considered along with hormonal treatment in patients with 1–3 +ve LNs. Patients with a low (RS < 18) recurrence risk and 1–3 +ve nodes are recommended to be on endocrine monotherapy. To note, clinicians should be aware that the optimal RS cut-off (<11 vs. <18) is yet to be determined for both RoR besides estimation of CT value (43, 44).

### MammaPrint and BluePrint

Before discussing BCA molecular subtypes, it is worth noting that almost 70% of all BCA cases have HR +ve and Her2 –ve tumor characteristics (45–47). The remaining 30% are split into either triple -ve or Her2 +ve clinical subtypes. Even though there is no consensus, the most common comprehensively recognized BCA classifications are luminal-type, basal-type, and Her2-type which represent tumors having HR +ve, triple -ve and Her2 +ve, respectively (47, 48). For the purpose of this review, we will be referring to BluePrint 80-Gene as Amsterdam 80-gene and MammaPrint 70-gene, Amsterdam 70-Gene as MammaPrint from this point onward.

#### MammaPrint

##### General Information

MammaPrint (Agendia, Amsterdam, The Netherlands) which is also known as “Amsterdam 70-gene prognostic profile” was the first In Vitro Diagnostic Multivariate Index Assay to be cleared by the FDA in 2007 (49). It was the first molecular diagnostic kit with a randomized prospective clinical trial endorsing its clinical utility (49, 50). It is validated for use in stage I and II disease irrespective of ER/HER2 status, with a tumor size ≤5 cm, and 0–3 +ve LN (51, 52). It is heavily mentioned in the guidelines of AJCC 8th edition, ASCO, NCCN, ESMO, and St Gallen’s, with an evidence level of IA (12). It was established using a “DNA microarray analysis of gene expression arrays on frozen tissue”, but it has now been amended for usage with FFPE tissue (12, 53, 54). Again, a mathematic model is used to calculate a score that stratifies BCA patients based on prognosis (8). Around 25,000 genes were included in the original training set. These were evaluated utilizing archived frozen-tissue samples from 78 patients aged <55 years with LN –ve tumors <5 cm in diameter (55). This assay describes the tumor expression profile, which entails 70 genes involved in the six well-defined hallmarks of cancer. While most of the genes enlisted have well-described biological functions, remaining genes are postulated to have an integrative role in BCA biology. These genes fall into two broad categories: 1-apoptosis evasion genes, which include BBC3 and EGLN1, contributing to acquired resistance to apoptosis, and FLT1, HRASLS and many others, contributing to proliferation and oncogenic transformation-2- genes. Overexpression of these genes leads to insensitivity to anti-growth signals which includes TGFB3, disrupting antigrowth signaling, RASSF7, DCK and MELK, increasing proliferation and oncogenic transformation (56). Other genes include those involved in unlimited replication ability, tissue invasion and metastasis and continuous angiogenesis as well as in epithelial-mesenchymal transition (56). It is important to mention that none of the hormonal receptor genes (ER, PR, and Her2) or proliferation gene (Ki67) were included in this genomic assay (55). Sparano and colleagues suggested the so-called rule of 4 for CRs i.e., a risk is considered high if the tumor has a “total of 4” (35). A tumor having a size more than 4 cm and a grade of 1, a size more than 2 cm and a grade of 2 or a size more than 1 cm and a grade of 3 are all considered as high CR (29, 35). The CR was defined low if the high-risk criteria were no met (35). In patients with high CR, HR +ve, HER2 -ve BCA and no or limited (one to three) involved LN; MammaPrint can be used in decisions regarding withholding CT (5, 6, 11). Patients with low CR are unlikely to gain from CT treatment irrespective of the results of this assay (8, 11). For those with LN involvement and a low risk by Amsterdam genetic profile, counseling should be provided to highlight that the chemotherapeutic benefit cannot be omitted, especially in patients with >1 involved LN (8, 11).

##### Validation Studies

The Netherlands Cancer Institute fresh-frozen tumor bank was the first to use this assay. Samples from 295 patients aged ≤52 years, split into 51% node -ve or 49% +ve disease, were examined. One hundred twenty patients out of 144 from the LN +ve cohort received adjuvant therapy while only 10 out of the 151 LN -ve patients received prior treatment (11). Adjuvant therapy in this study consisted of mainly of CT alone or CT with hormonal therapy. The distant disease-free survival was 50.6 and 85.2% in the groups with a poor-prognosis signature and good-prognosis signature, respectively (11, 57). Buyse et al. later conducted a validation study in 307 patients as part of the TRANSBIG consortium (58). They found out that this assay adds independent prognostic information, including time to distant metastasis and OS, to clinicopathologic risk assessment for patients with early BCA (59). Patients in the high-risk group had a lower 10-year OS when compared to the low-risk group (69 vs 89%) (58). Other studies confirmed the prognostic value of the MammaPrint, such as the “microarRAy-prognoSTics-in-breast-cancER RASTER” study (52). In this prognostic study involving 427 patients, treatment decisions were based on standard guidelines, the MammaPrint, and patient and doctor preferences. Out of the total enlisted patients, 124 patients were considered low risk by MammaPrint, but high risk according to other factors such as tumor size, age, nodal status, and other pathological and clinical factors. Of these patients, 76% did not receive adjuvant CT, and 98% survived with no disease recurrence in 5 years. Thus, using the low-risk gene signature result to decide on withholding CT did not negatively affect rate of recurrence (52).

##### Role in Node Positive Disease

MammaPrint has been validated in several studies involving multiple LNs as well. Mook et al. proved that the 10-year distant metastasis-free survival (DMFS) was 91% and BCSS was 96% for the good prognosis group vs. 76% for both DMFS and BCSS in the poor prognosis group (60). In addition, BCSS was significantly predicted using MammaPrint with a multivariate hazard ratio of 7.17 (95% CI 1.81 to 28.43; P = 0.005) (60). Another study done by Saghatchian involving 4–9 LN +ve 173 patients, revealed that the 5-year DMFS (P < 0.01) and 5-year OS (P < 0.01) was 87 and 97% in the good prognosis group and 63 and 76% in the poor prognosis (61).

The largest trial involving MammaPrint was the MINDACT trial which stands for Microarray In Node negative and 1–3 +ve LNs Disease may Avoid Chemotherapy. It included 6,693 women with invasive BCA as proven by histology, 0- +ve nodes, and no distant metastasis. Initially, only females without any +ve nodes were included in the trial, and in 2009 the study was amended to be inclusive of patients with 1–3 +ve LNs as well. The genomic risk (GR) of each patient was assessed by using the MammaPrint assay, while using a modified version of Adjuvant! Online to determine the corresponding CR (62). No CT was administered to patients with low genomic and CRs, while administering CT to those at high genomic and CR. In the group with discordant genomic and CRs, patients were randomly assigned to CT vs no CT groups. Hormonal therapy was recommended for 7 years in all groups based on the hormonal status. The primary analysis of the study was assessing the distant metastasis-free survival (DMFS) in female patients with an increased risk clinical features but decreased risk genomic profiles not receiving CT (63). The primary end point of the trial was met, as the inferior margin for DMFS at 60 months was 92.5% in the targeted subjects, which exceeded the 92% pre-specified threshold. Using this assay, 46.2% of patients with a superior risk clinically would not receive CT, while having a 1.5 and 2.1% increased risk of distant metastasis in the intention to treat population and in the per-protocol analysis respectively. However, the trial was not powered to omit the advantage of CT and would require extensive follow-up for female patients with LN +ve disease (28). In the node +ve subgroup, the absolute value from CT in terms of DMFS was 0.7%. These patients had a high clinical/low GR. As such, the MINDACT trial provided restricted backing for the use of MammaPrint in node +ve disease, and the benefit of chemotherapeutics could not be excluded due to insufficient power (28). As per ASCO, the MammaPrint assay can be used while deciding to withhold adjuvant CT in HR +ve, LN -ve patients, as well as in select patients with +ve LN. The clinical utility was only evidenced in patients at high CR (63). In patients with ER/PR +ve, HER2 -ve BCA with 1–3 +ve nodes and at increased CR, the MammaPrint assay may be used to decide on deferring adjuvant treatment consisting of systemic CT. This can be done after informing the patient that a benefit of CT cannot be excluded especially if more than one LN is involved (63). No evidence is available to support the use of MammaPrint to decide on adjuvant systemic CT in patients with HER2 +ve BCA as well as in patients with triple -ve BCA (64). A *post hoc* analysis of the MINDACT trial presented at the 2019 San Antonio Breast Cancer Symposium showed that patients with premenopausal BCA and discordant genomic and CRs not receiving adjuvant CT had a small increase in distant metastasis. There was an absolute difference of about 3% in the 5-year DMFS in women ≤50 years with high clinical yet low GR as per the MammaPrint assay (93.1 vs 96.1%). On the other hand, women >50 years did not show a difference in the 5-year DMFS rates with or without adjuvant CT (95.4 vs. 95.2%) i.e., a benefit to CT could not be demonstrated (65). Similarly, patients with a low CR and a high GR had no difference in 5-year DMFS when assigned treatment based on CR or GR i.e., no CT, 95% vs with CT, 95.8% respectively (51). Here it is important to mention that panellists from BCTEG compared suggestions from the Early Breast Cancer Trialist’s Collaborative Group (EBCTCG) to the suggestions that a 10-year follow-up for the MINDACT is needed. The EBCTG proposes that most benefit from chemotherapeutic treatment concerning 20-year recurrence risk occurs early on in the progression of the disease (66).

Another prospective study, the “Prospective Study of MammaPrint in Breast Cancer Patients With an Intermediate Recurrence Score, PROMIS trial” was performed to assess how the treatment decision changed before versus after receiving the MammaPrint result. Patients with early-stage disease totalling eight hundred forty patients were enrolled. These patients an intermediate 21 gene assay recurrence score (RS) of 18–30. Using MammaPrint, 44.5% were classified as low risk, while 55.5% as high risk. 90.6% of decreased risk diseased females were suggested no adjuvant chemotherapeutic treatment, and 87.8% of increased risk patients were suggested adjuvant chemotherapeutic treatment. The nodal status did not affect the change in treatment decision. 33.7% of participants with LN -ve disease and 32.1% of patients with LN +ve disease had an alteration to their CT management decision. The pitfalls of the PROMIS trial were the lack of survival and recurrence data, and having inclusion criteria limited to patients with ER +ve/HER2 -ve disease (67). In the sub analysis of the PROMIS trial presented at the San Antonio Breast Cancer Symposium, ovarian function suppression and ET were suggested as an alternative for women less than 50 years with low CR (as assessed by the MINDACT, modified Adjuvant Online! Algorithm) and an RS of 16–25. Almost 46% of women less than 50 with an RS of 21–25 were found to be at low risk as per MammaPrint and can avoid CT as per the MINDACT trial data. MammaPrint can safely identify 20–60% of women less than 50 with an intermediate RS of 18–30 as low GR with excellent survival with ET alone (68).

Finally, it is worth noting ASCO does recommend the use of MammaPrint 70-gene assay in ER/PR +ve, HER2 -ve, LN +ve (1–3 LNs) BCA patients who are at high CR since patients’ benefit of CT cannot be excluded. ESMO considers MammaPrint and Oncotype DX, both first-generation signatures, as prognostic for ER +ve, Her2 -ve tumors. Neoadjuvant CT is indicated if there is a high risk or score. Concerning MammaPrint, there was no supplementary advantage for adjuvant chemotherapeutic treatment in females with BCA at increased CR and decreased GR. The 2019 ESMO guidelines endorsed a utility of panels such as MammaPrint in conjunction with clinical and pathological factors to lead systemic treatment decisions in challenging patients such as HER2 -ve and node -ve or with one to three +ve LNs (69). NCCN clearly states that for patients with a low RoR based on PAM-50 who were treated with ET alone have a DR risk of <3.5% and no DR at 10 years (44).

#### BluePrint

##### General Information

Another genomic assay is the BluePrint 80-Gene Molecular Subtyping Test which was developed by Agendia, Amsterdam, the Netherlands to measure the expression of 80 genes. These genes are related to functional pathways and help determine the intrinsic BCA molecular subtypes. Bridging research molecular subtyping and clinical pathology, this assay works by computing RNA expression extracted from FFPE from breast tumor tissue (48). The agreement between immunohistochemistry confirmed receptor status and molecular subtypes highlights the role molecular profiles play as oncogenic processes; these processes are driven by pathways where ER, PR, and Her2 play a major role (70–73). For that reason, it was postulated that differences in clinical outcomes between previously discovered subtypes of BCA would be uncovered by Amsterdam 80-gene (45). In this assay, a tumor’s similarity to three sets of genes split into 58, 28, and 4 genes corresponding to Luminal-type, Basal-type, and Her2-type representative profile respectively is obtained (48). The test result would be the subtype with the most +ve magnitude (48). Based on functional molecular pathways, this assay computes mRNA levels of the previously mentioned genes to classify between three BCA subtypes: luminal, HER2, and Basal (45).The luminal group can be further stratified into luminal A or luminal B by the Ki67 fraction or by MammaPrint, which is superior for making this differentiation (74). Luminal A is defined as having a Ki67 <14% or being MammaPrint low-risk, and luminal B is defined as having a Ki67 expression ≥14% or being MammaPrint high-risk (45, 75).

##### Validation Studies

In 2011 Krijgsman et al. used 6 different studies with a total of 1,212 patient specimens to identify the total number of genes (N = 80) that best differentiated the three molecular subtypes described above (45). Immunohistochemistry for ER and PR along with chromogenic *in situ* hybridization for Her2 was utilized to detect protein levels. TargetPrint assay was used to measure all three receptors’ mRNA levels respectively (45). It was revealed that the RNA-based Amsterdam 80-gene can better categorize tumors and accomplish a superior response stratification than IHC-based clinical subtyping (45, 51, 74, 76, 77).

Even though most tumors show a single functional subtype, Ellappalayam et al. showed that a dual Amsterdam 80-gene subtype was displayed by a small proportion of samples (78). Differential expression analysis (DEA) proved that these dual subtypes have particular genomic aspects which ameliorate their therapeutic recommendations (78). For example, DEA done on samples of patients identified by the Amsterdam 80-gene as dual luminal B/Basal subtype revealed an upregulation of the genes AGR2, an indicator for poor outcome, and ESR1, an indicator for therapeutic resistance (78–80).

Mittempergher et al. recently discovered that this assay had a repeatability and precision of 99.0 and 98.3% respectively when followed over 3 years (48). Over the same period, this assay also had a 98 and 99% concordance for reproducibility over-time and reproducibility under different conditions, respectively (48). Concerning pathological complete response to neoadjuvant therapy, basal-type and Her2-type tumors were more sensitive to CT than luminal-type tumors (45, 74, 81, 82). On the other hand, luminal-type tumors had a more encouraging distant metastasis free survival out of all three subgroups (74, 82).

In a study done by Viale et al. using the same patient population enrolled in the previously mentioned MINDACT trial, IHC/FISH was compared to both Amsterdam 80-gene and Amsterdam 70-gene (83). In this study, around 30% (N = 1738) of tumors were reclassified after molecular subtyping was applied to tumors identified by pathological subtyping, IHC/FISH. Interestingly, in this study Ki67 was used as well as part of the pathological subtyping. Out of all the tumors identified as luminal B by IHC/FISH, 54% were identified as luminal A by molecular subtyping. Similarly, out of all the tumors identified as Her2 +ve by pathological subtyping, 38 and 5% were classified as luminal (A and B) and basal-type respectively (83).

##### Role in Node Positive Disease

In the WSG-PRIMe study, which stands for West German Study Group-PRospective Study to Measure the Impact of MammaPrint on Adjuvant Treatment in Hormone Receptor-positive HER2-negative Breast Cancer Patients, 452 HR +ve/Her2 -ve with up to three involved LNs were recruited, and 63.5% of patients were assigned to the low-risk category (84). Almost 15.1% of the patients were switched into the CT intention group after they were not supposed to receive CT, and 14.0% of patients did not receive CT after they were supposed to receive CT (84). Last, around 70.9% of the patients the treatment recommendation did not change before and after MammaPrint (84). In this study, there was a 65.3% concordance between IHC assessment and Amsterdam 80-gene subtyping. Using BluePrint, CT recommendations were strongly associated with molecular subtype in 94.1 and 92.3% of luminal B patients and luminal A patients respectively (84).

Data from the APHINITY, Adjuvant Pertuzumab and Herceptin IN Initial TherapY in Breast Cancer, trial includes 4,805 patients with histologically confirmed Her2 +ve early BCA treated with standard adjuvant CT and trastuzumab plus either pertuzumab or placebo for 1 year (85, 86). Half of the cancers were classified as luminal subtype, 28 and 22% were identified as Her2 subtype and basal subtype, respectively. While the vast majority of luminal cancers were single activated, approximately half of Her2 activated cancers were identified as dual, and 40% of basal cancers were dual activated. When followed up for 45 months, the IDFS was significantly beneficial for adding pertuzumab to CT and trastuzumab at 3 years with a hazard ratio of 0.81 (95% CI, 0.66–1.00; P = 0.045) (85). These findings were similar to the results obtained by Minckwitz et al. (86). Among the APHINITY samples (n = 969) usable for Amsterdam 80-gene subtyping, patients having Her2 +ve tumors with a single activated Her2 subtype had a greater benefit with the addition of pertuzumab to CT/trastuzumab than any other tumor subtypes. On the other hand, Krop et al. found that patients having Her2+ve BluePrint single-activated Basal-type tumors had a significant worse prognosis HR 1.67 (95% CI, 1.11–2.51) (85). Last, after undergoing a multivariate analysis it was revealed that nodal status was only associated with IDFS: HR 4.53 (95% CI 3.10–6.62) for functional the basal subtype, HR 4.51 (95% CI 3.09–6.58) and HR 4.49 (95% CI 3.08–6.56) for the luminal and Her2 subtypes, respectively (75).

In a study evaluating the role of both MammaPrint and BluePrint have on dictating luminal BCA treatment, Wuerstlein et al. found that a switch in CT recommendation occurred in 29.1% of the cases (84).

Finally, in an effort by Whitworth et al. to predict chemosensitivity, by pathologic complete response (pCR), or endocrine sensitivity, by partial response, a total of 426 patients with histologically proven BCA were recruited as part of the Neoadjuvant Breast Registry Symphony Trial (NBRST) (74). In this study, 94 tumors were reassigned to different categories using Amsterdam 80-gene compared to what they were initially assigned in using conventional methods, IHC/FISH. Out of 403 patients receiving neoadjuvant CT and 20 patients receiving neoadjuvant ET, 25% achieved pCR and 65% achieved partial response respectively. Similar to the findings in Cortazar et al. where patients identified as HR +ve and Her2 -ve had a pCR rate of 8% (grade 1 and grade 2), patients with tumors having similar characteristics in this study had a rate of 10%, and patient with tumors identified as luminal A and luminal B had a pCR of 2 and 7%, respectively (74, 87). When comparing the pathological complete response to neoadjuvant CT, Amsterdam 80-gene Her2 +ve patients had a superior rate (53 vs. 38% p = 0.047) than IHC/FISH Her2 +ve patients (74).

### PAM50

#### General Information

The Predictor Analysis of Microarray 50 (PAM50, by NanoString Technologies, Seattle, WA) was developed using microarray and quantitative RT-PCR on FFPE tissue (88). Approved by the FDA in 2013, PAM50 is a 58-gene test that describes an individual tumor by intrinsic subtype and categorizes BCA samples into one of the following subtypes: luminal A, luminal B, Her2-enriched, and basal-like (5, 16, 89). It also quantifies the rale of proliferation, luminal gene expression, ESR1, PGR, and ERBB2 expression (90, 91). It is incorporated in the ESMO, St Gallen’s, NCCN, ASCO, and AGO guidelines and has an evidence level of IIA (4, 15). PAM50 results are used to generate the RoR score. This assay, used mainly with postmenopausal females, is used to approximate the risk of DR within 10 years of diagnosis of early-stage, HR +ve disease with 1–3 +ve LN after 5 years of ET (11). Based on these activity levels, RoR score in node -ve cancers are classified as intermediate (41–60) with scores at the opposite extremes of this range classified as low (less than 41) and high (more than 60) risk (92). RoR score in node +ve cancers are classified as low (0–40) or high (41–100) risk (5, 11, 92). Concerning node +ve ER/PR +ve, HER2 -ve BCA ASCO states that more data is needed to determine whether this assay can be utilized in guiding the use of adjuvant CT (93). The prognostic capacity of RoR seems to outperform that of RS, hence more precisely identifying low vs high-risk groups and adding prognostic information in patients with node +ve disease.

#### Validation Studies

Some studies have revealed Prosigna’s ability in prognosticating recurrence risk. Around 1,906 genes extracted from 218 patients, 29 breast biopsy samples and 189 malignant breast samples were included in the training set for Prosigna (90). Fifty genes were then chosen based on their involvement in distinguishing the different subtypes. Next, tumor samples from 894 patients were investigated. Out of these patients, 710 patients had LN –ve BCA and didn’t receive any therapy, and 133 patients received CT (94). A difference in recurrence free survival was observed; HR relative to luminal A was 1.33–1.79 for patients with basal-like disease, 2.53–3.25 for patients with her2-enriched disease, and 2.43–2.88 for patients with luminal B disease (90). A further validation study done by Nielsen et al. on 786 females with ER +ve disease receiving tamoxifen for 5 years (95). These patients had either LN +ve or LN –ve disease. RoR scores was a prognostic indicator of recurrence free survival in 96.7, 91.3, and 79.9% of patients in the low-risk group, intermediate-group and high-risk group respectively (95).

#### Role in Node Positive Disease

The two main retrospective studies that further validated the use of PAM50 are the “Austrian Breast and Colorectal Cancer Study Group 8” (ABCSG-8) trial and the “Arimidex, Tamoxifen, Alone or in Combination” (ATAC) study (96, 97). The ABCSG-8 enrolled more than 3,700 postmenopausal women aged 80 or below and randomly assigned them to receive either tamoxifen for 5 years or to 2 years of tamoxifen followed by 3 years of anastrozole. Data recently published from this trial, along with data from a study on 1,478 post-menopausal women from this trial concluded PAM50’s clinical validity for predicting the risk of disease recurrence with a level 1 evidence. What is more interesting is that a 10-year risk of metastasis of less than 3.5% in the group with low RoR makes additional CT an unwarranted overtreatment. This study showed that even among some node +ve BCAs, some patients have a negligible risk of metastasis and could be spared CT if these results were confirmed (98). The ATAC trial proved that the clinical treatment score was the strongest prognostic score for late DR, and that the RoR score contributed to a much greater extent than the Oncotype RS (99). A similar study was conducted by the Danish Breast Cancer Group on postmenopausal women with a 1 to 3 +ve LN or LN -ve patients with either a tumor grade of 2–3 or size >2 cm and was able to prove the prognostic value of the RoR score (100). They performed a retrospective tumor analysis on recruited patients who were treated during 1999–2003 with hormonal therapy alone. More than 2,500 blocks were collected and 1,480 blocks were from node +ve patients (11). In this study, PAM50 identified at least 37% of patients with 1 +ve node who have a favourable prognosis and may be spared adjuvant CT clinically (100). These patients had a DR rate less than 5% at years (100).

This assay is now being evaluated by the OPTIMA (“Optimal Personalized Treatment of early breast cancer using Multi-parameter Analysis”). The latter is a randomized trial, which is expected to provide data on managing patients with increased CR BCA (ER +ve, HER2 -ve BCA with 1–9 +ve nodes or tumor size >30 mm). In the OPTIMA trial, patients are randomized to receive either chemotherapeutic therapy or chemoendocrine therapy for patients with RoR >25 vs hormonal treatment alone for those with RoR ≤25 (15). What is more, the OPTIMA preliminary study (OPTIMA prelim) was partially blinded randomized control trial with adaptive design involving 35 UK hospitals. In this trial, 313 patients were randomized into two groups: a standard group, where patients would receive chemo-endocrine therapy, and a test-directed group, where patients would receive CT is RS >25 (101–103). This prelim trial was important for several reasons. First, even though an optimal test is yet to be recognized, it portrayed that molecular testing is cost-effective with a probability of 86% along with a quality-adjusted life-year gains (range 0.17–0.20) (102). Interestingly, it revealed that only small differences in costs and quality-adjusted life-years existed between Oncotype DX, MammaPrint, PAM-50, MammaTyper, IHC4, and IHC4-AQUA (102). Second, based on its findings, OPTIMA was deemed appropriate to take place (103). Third, OPTIMA prelim showed that Oncotype DX quantified the highest proportion of tumors as low risk [82.1%, 95% (CI) = 77.8 to 86.4%] when compared with PAM50, IHC4, MammaPrint or and IHC4-AQUA (101). Intriguingly, when comparing these five tests’ ability to stratify patients into risk groups, it was revealed that these multiparameter tests provide broad equivalent risk information (101). Concerning subtype classification, the trial revealed that there was discordant subtyping in 40.7% (N = 123) of tumors when using Blueprint, MammaTyper, and PAM50 (101)

Here again, it is worth noting that ASCO does not recommend the use of PAM 50 RoR score in patients with ER/PR +ve, Her2 -ve, node +ve patients.

### EndoPredict

#### General Information

Another genomic assay is EndoPredict (Myriad Genetics Salt Lake City, UT) which utilizes RT-PCR of 12 genes, split into three entities. The first entity consists of eight cancer related genes. The second and third entity consist of three RNA reference genes, and one DNA reference gene (104). EndoPredict is used to calculate the endopredict (EP) risk score, which is used along with tumor size and nodal status to calculate the EPclin score, a comprehensive risk score. [3] It can be run using either FFPE samples, tissue from the diagnostic core biopsies, or from the final surgical specimen (31). It is incorporated in the guidelines of ESMO, St Gallen’s, ASCO, and AGO (89). It is beneficial in the identification of a subsection of females with ER +ve, HER2 -ve tumors that are either node -ve or have 1–3 LNs, and that have a very low RoR without adjuvant CT. It also appears to identify patients at low risk for a late recurrence. An EPclin Risk Score higher than 3.3287 is deduced as the disease having a high RoR (8). In general, Endopredict is used to guide treatment decisions for both CT and anti-hormonal therapy (12).

#### Validation Studies and Role in Node Positive Disease

Three studies were mainly used as clinical evidence for the use of this signature. The first study is the GEICAM trial, which stands for Grupo Español de Investigación en Cáncer de Mama (105). The EP score and the EPclin score were obtained in 555/800 available samples (89). Based on EP, one fourth of the patients were classified as low risk. Metastasis-free survival was 93 and 70% in the low-risk group and the high-risk group, respectively. Based on EPclin score, the low-risk group constituted 13% of patients and these had excellent outcomes and no DR events (89). This proved that EP is a non-dependent prognostic factor in node +ve ER +ve/HER2 -ve diseased patients treated with adjuvant chemotherapeutic treatment followed by endocrine treatment (30). The two other studies were the ABCSG-6 and ABCSG-8 trials. More than 1,700 postmenopausal patients were treated with 5 years of hormonal therapy alone, and it was also proven that both EP and EPclin could be used as reliable tools. EP provided reliable prognostic information for identifying early and late distant metastasis better than combining clinical parameters. Similarly, using EPclin, a subdivision of patients was identified to have an excellent long-term prognosis after only 5 years of exclusive ET (106). The 10-year DRR was 4% in the EPclin low risk patients and 28% in the EPclin high-risk patients in the ABCSG-6 (P < 0.001). Similarly, the 10-year DRR was 4% in the EPclin low risk patients and 22% in the EPclin high-risk patients in the ABCSG-8 (P < 0.001) (107). Even though these studies have provided promising preliminary results for the use of Endopredict in node +ve patients, ASCO considers the level of evidence to be insufficient to make a strong recommendation (12).

Lastly, ASCO states that EndoPredict 12-gene RS should not be used in node +ve BCA. ESMO states that second-generation signatures such as Prosigna and Endopredict are prognostic when evaluating ER +ve, Her2 -ve tumors along with tumor size and nodal status. These signatures can be carried out in biopsy or surgical specimen and neoadjuvant CT is indicated if there is a high risk or score. Similarly, the 2018 San Antonio Breast Cancer Symposium recommended that EPclin is prognostic for early (0–10 years) and late (5–15 years) DR (108). NCCN states that patients enrolled in the transATAC study with an EPclin score <3.33, i.e., a low recurrence risk, have a 5.6% risk of DR at 10 years (109).

### Breast Cancer Index

#### General Information

The Breast Cancer Index (BCI) by Biotheranostics, Inc. San Diego, CA is a mix of two profiles: the antiapoptotic homeobox B13-to-interleukin 17B receptor expression ratio (H:I ratio), representing a 2-gene ratio, and the Molecular Grade Index, representing five proliferation genes (15). This score was developed to determine the probability of benefiting from extended adjuvant ET in postmenopausal patients with ER +ve, LN -ve disease (20). It estimates how likely late recurrence takes place i.e., 5 to 10 years after diagnosis with scores ranging from 0 to 10 (5, 8, 110). Malignancies with scores <5 are categorized as having low risk of late recurrence (110); a score more than 5.1 corresponds to having a high risk of late recurrence (31, 110). The BCI Predictive score also “estimates how likely a woman is to benefit from taking hormonal therapy for 5 more years for a total of 10 years; the results are reported as either low likelihood of benefit or high likelihood of benefit (5, 110)”.

#### Validation Studies

This molecular signature obtained its clinical validation and indication of prognostic utility from two trials: the ATAC trial and the Stockholm trial. The former trial provided prognostic utility through a blinded retrospective analysis of more than five hundred eighty patients (111). These ER- +ve patients were randomized into an untreated arm and another arm treated with tamoxifen. DR risk was successfully predicted using HOXB13:IL17BR (H: I) and molecular grade index (MGI) (111). Several studies since have further validated its use. The MA.17 trial has validated its predictive ability as a biomarker by studying the use of letrozole as an extended adjuvant therapy. In patient receiving ET, there was a 10% reduction in recurrence (P = 0.007) in patients having a high H/I ratio compared to no statistically significant difference in patients with a low H/I ratio (112). In addition, BCI has been shown to have a partial absolute disease-free survival with the use of tamoxifen, 2–4.7%, along with known side effects from toxicity (112). With that being said, BCI can be used to check who is most likely to benefit from extended adjuvant hormonal treatment compared to those who could be spared since they will not gain an advantage from extended adjuvant hormonal therapy (113–115).

#### BCI vs. Multiple Genomic Assays

Some studies have described the role of BCI to estimate the risk of late recurrence compared to other genomic assays. In a study involving 774 postmenopausal patients with ER +ve Her2-negtive BCA, the prognostic value of six signatures including RS, RoR, BCI, EPclin, Clinical Treatment Score (CTS), and IHC4 were compared (116). Similar to the study above, LR statistics were utilized with the χ2 test. CTS is based on nodal status, tumor size, grade, age, and endocrine treatment (117). RoR followed by BCI and EPclin were the genomic expression profiling assays providing the most prognostic information overall having Hazard Ratios of 2.56; 95% CI, 1.96–3.35, 2.46; 95% CI, 1.88–3.23 and 2.14; 95% CI, 1.71–2.68, respectively (116). These signatures provided the most prognostic information for late DR in 774 patients with node -ve disease (116). Even though less information was available for all six signatures in 183 patients with 1–3 +ve nodes, BCI (LR-Δχ2 = 9.2) followed by EPclin (LR-Δχ2 = 7.4) provided the most prognostic information (116). Sestak and colleagues proved that this combination, molecular factors and clinical features, was more revealing, especially for patients with LN +ve disease (116). Nevertheless, restricted independent data was presented from any test in patients with node +ve disease and that is why further studies involving these patients are needed. Second, LRs do not provide any clinical utility preferring any test; LRs only specify statistical contrasts among the tests (116).

In another prospective study done by Sgroi et. al., tumor blocks retrieved from the archives of 665 patients enlisted in the transATAC trial were compared using three genomic assays, including BCI, in predicting late DR (9). The specimens used belonged to the ER +ve postmenopausal patients with node -ve BCA. Assessing the prognostic ability of BCI for DR over 10 years, two BCI models, cubic and linear, were used; also, LR-Δχ2 from Cox proportional hazards models was utilized (9). In this study, BCI-L, linear model, was the lone significant prognostic test for both early (0–5 years) and late (5–10 years) DR when compared with BCI-C, cubic model, IHC4 and 21-gene recurrence score (BCI-L Hazard Ratio 1.95 along with a 95% CI 1.22–3.14) (9). After undergoing a multivariable analysis, BCI-L, RR, and IHC4 had significant prognostic ability for only early, 0–5 years DR having Hazard Ratios of 2.77, 1.80, and 2.90 along with LR-Δχ2 = 15.42, LR-Δχ2 = 18.48, LR-Δχ2 = 29.14 (p < 0.0001), respectively (9). These findings suggest that BCI-L could be used to identify patients at high risk for late DR who would benefit from extended ET.

#### Role in Node Positive Disease

Some studies have even evaluated use of BCI in ER+ BCA patients who are node +ve with 1–3 +ve LN. BCI gene expression along with tumor size and grade were integrated into a novel Breast Cancer Index model (BCIN+) (118). This model classified 20% of patients out of a total of 402 with one to three +ve LNs as low risk with a 15-year risk disease recurrence rate of 1.3%. It was concluded that extended endocrine treatment may be spared in this subset of patients (118). This allowed patients to have a more individualized approach and to be spared the side effects of an additional five years of ET.

In a study done by Sgroi et. al., CTS and BCIN+ were compared in BCA patients with one to three +ve nodes. Out of 249 patients, 160 received up to 5 years of ET and 197 patients received adjuvant CT. While BCIN+ classified patients into two subsets, CTS classified patients into three risk groups. The former classified 77% of patients as high-risk with a 16.1% risk for late DR vs. those classified as low risk with 1.3% risk for late DR [HR 12.4 (1.7–90.4), p = 0.0014] (119). The latter classified patients into low-risk (29%), intermediate-risk (37%) and high-risk groups (34%) with a 4.2, 10.6, 22.1% risk for late recurrence, respectively (119). In a subset of patients who received 5 years of ET (N = 223), the former acknowledged around 20% of patients as low risk having a late DR rate of 2.1%. On the other hand, using CTS patient identified as low-risk and intermediate risk comprised 29 and 37% having a late DR rate of 5.2 and 10.3%, respectively (119). Based on these findings, it may be advisable that patients identified as low risk with BCIN+ have extended ET omitted from their treatment regimen to avoid unintended side effects.

In the Adjuvant Tamoxifen—To Offer More? (aTTom) trial, 583 patients with HR +ve, node +ve disease were analyzed to check the interaction between extended ET and BCI (120). Approximately half of these patients were classified as BCI-High. These patients derived a significant benefit from 5 extra years of tamoxifen treatment with a 10.2% absolute risk reduction based on recurrence-free interval, P = 0.027 and a hazard ratio of 0.35: 95% confidence interval 0.15–0.86 (120). On the other hand, BCI-low patients showed no significant benefit from 10 years versus 5 years of tamoxifen treatment with a negative 0.2% absolute risk reduction (P = 0.768) and a hazard ratio of 1.07 along with 95% confidence interval 0.69–1.65 (120). In this trial, continuous BCI levels emphasized the degree of advantage from protracted tamoxifen while centralized ER and PR did not (120). Finally, the interaction between extended tamoxifen treatment and BCI was statistically significant (P = 0.012) (120). More ongoing studies with node +ve patients are needed to guide combined clinical-GR assessment.

Here again, it is worth noting that ASCO does not recommend the use of the BCI should not be used in node +ve BCA despite emerging evidence for its use in LN +ve patients. Second, NCCN guidelines concerning BCI are based on a secondary analysis of the aTTom trial (44). Patients with both receptor and node +ve disease with a high H:I ratio had a significant advantage from extending tamoxifen therapy to 10 vs. 5 years (44). On the other hand, BCI low patients derived no benefit from extended adjuvant therapy (120). Last, both ASCO and NCCN support the use of BCI as a prognostic marker and not as a predictive marker for extended adjuvant ET (58, 121).

### Genomic Grade Index

#### General Information

The genomic grade index (GGI) is a gene expression signature of 97 genes that were found to best distinguish histologic grade 1 (i.e., low grade, well-differentiated) from grade 3 tumors (i.e., high grade, poorly differentiated) (31). This assay is frequently utilized after many inconsistencies were found in grading among pathologists concerning the histological grade of breast tumors in an effort to improve prognostication (16). This assay uses an RT-PCR version that can also use FFPE samples (31). It splits grade II (i.e., intermediate grade) ER+ BCA into high or low grade categories and therefore confers significantly different prognoses on otherwise similar tumors (5, 8, 31, 122). Not only is high GGI related to decreased relapse-free survival in patients who do not go on to receive adjuvant CT, but it also linked with increased sensitivity of response to neoadjuvant CT in both ER -ve and ER +ve patients (15, 31).

#### Validation Studies and Role in Node Positive Disease

One of the earliest validation studies done by Sotiriou et al. included 597 cancerous tumors samples. The relationship between gene-expression (GE) profiles of primary breast tumors and DRFS was observed along with the patterns of GE among the different grades of disease (123). Based on a training set of 64 ER +ve tumors, GGI was defined as low in the presence of low-grade tumors, a -ve GGI corresponds to a GE grade of 1 (low grade) and finally a +ve or 0 GGI corresponds to a GE grade of 3 (high grade) (123). Among the intermediate-grade ER+ve patients treated with tamoxifen, those with a high-genomic-grade subgroup had an adverse disease outcome (122). Last, among patients having grade 2 tumors, those with a higher GGI had a higher RoR (HR 3.61; 95% CI 2.25–5.78; P < 0.001) (122).

A validation study published by Loi et al. showed a 10-year distant-recurrence-free survival (DRFS) in GGI of 83% in ET-treated patients irrespective of nodal status (122). A small preliminary biomarker study auxiliary to the PACS01 trial compared GGI to histological grade, mitotic activity index, Ki67 immunohistochemistry status, and mRNA levels in LN +ve diseased patients treated with adjuvant anthracycline-based chemotherapeutic treatment (124). GGI exhibited the strongest correlations in different aspects. GGI had a superior DRFS than the other four parameters in more than 200 patients and in the 95 histological grade 2 patients. GGI, Mitotic Index (MI) and Ki67 all have a prognostic importance in invasive disease (125). The 5-year DRFS in a small cohort of node +ve CT treated patients was 89% compared to 64% for patients with GG1 and GG3, respectively. Two other prospective studies have further supported GGI’s use in node +ve disease (126). In the BIG 1-98 trial, the GGI served as a good predictor of relapse depicted as a directly proportional increase of 11% in hazard rate for each 10-unit increase in GGI. On the other hand, for node -ve diseased patients, no statistically significant difference was found in the risk of relapse between high and low GGI (127). The WSG EC-Doc study has also showed similar results (128).

Finally, using more than 200 tumor samples, the ability of GGI to predict a response to neoadjuvant CT was studied by Liedtke and colleagues. Approximately 73% of these patients (N = 167) had a +ve LN status (16). Among the GGI high-risk group, 44.8% (N = 60) had ER+ve disease; in total, there was 132 patients who had an ER+ve disease (129). In this study, neoadjuvant CT consisted of paclitaxel, fluorouracil, doxorubicin, cyclophosphamide, and the pCR was obtained using the residual cancer burden method (129). There were three significant findings from this study. First, there was a significant (P < 0.001) association between having an ER -ve disease and having a high tumor grade and GGI (129). Second, there was a correlation between having a high GGI and increased sensitivity to therapy (OR 1.86, 95% CI 1.15–3.00; P = 0.011). Last, after being followed up for a median of 28 months, patients with ER+ve disease and high GGI continued to have a poor prognosis after systemic therapy having a median DMFS If 67.5 months vs 93.2 months in patients having a low GGI (p = 0.005) (16, 129).

### Immunohistochemistry and Mammostrat

#### Immunohistochemistry

The immunohistochemistry (IHC4) assay incorporates a semi quantitative assessment of ER, PR, HER2, and Ki67 expression using IHC with clinicopathologic aspects into a multivariate model (5, 31). This assay is used to predict the risk of distant metastasis (31). The aforementioned markers are extensively used in the clinical setting to outline surrogate molecular subtypes (12, 31). As first defined, it utilizes FFPE specimen, can supposedly be achieved locally, and is a potentially cost-effective technique of refining prediction of early-stage disease with a validated recurrence risk signature (31). In a study of 84 patients, IHC techniques were compared to conventional histopathologic procedures. IHC for Epithelial Membrane Antigen detected metastasis of less than 2.0 mm in sentinel LNs in 36 patients, compared to histopathology, which detected metastasis in 32 patients. To note, two patients had +ve sentinel LNs on histology and were -ve on IHC. These two patients were later found to have poorly differentiated disease (130). One of the latest studies revealing its application in node +ve cancers revolved around devising a novel IHC using alternating current electric field (131). The importance of this study lies in the fact that determining axillary LN status is one of first steps to deciding whether adjuvant systemic therapy should or should not be administered. Intraoperative diagnosis usually consists of “one-step nucleic acid amplification and haematoxylin and eosin staining of frozen sections” (132). Greater number of metastasis could be detected if IHC is used. However, there are several setbacks for its use. First, the standard IHC protocol requires extensive time therefore it is not practical to complete intraoperatively. The previously mentioned novel IHC requires about only 20 min and easily overcomes this hurdle (131). Second, the results have no impact on patient outcome, systemic treatment, or radiotherapy. Even though this technique had a superior sensitivity, specificity and accuracy when compared to “longstanding” IHC, both tools had no added value on patient outcome and on whether or not to use systematic treatment or LN therapy. When compared to findings in the ATAC trial, IHC score was found to have similar results to Oncotype DX at a much cheaper cost (133). A total of 786 patients from the ATAC trial were evaluated for the IHC score. These patients were ER +ve and did not receive adjuvant CT. Even though it is much less expensive, IHC’s use is not without any challenges of its own. The utilization of this assay requires a joint effort to standardize the IHC methods in order to be able to measure Ki67 expression (133). Actually, Ki67 expression values and cut-offs for decision-making were not found to be standardized between different sites without finding a common scoring methodology (133, 134). This was proven by the International Ki-67 Breast Cancer Working Group of the Breast International Group and the North American Breast Group in 100 tumorous samples across eight laboratories (134). Reproducibility between labs was high, but reproducibility within the same lab was much lower (134).

#### Mammostrat

The “Mammostrat” test is composed of five antibodies that stratifies tumors as low-risk, moderate-risk, and high-risk (135). This kit is not considered a true genetic test (12). It is a prognostic test that utilizes the following markers: CEACAM5, HTF9C, NDRG1, SLS7A5, and TP53 and give insight into prognosis in tamoxifen treated patients (12). Four studies have already proved Mammostrat’s prognostic role for ER +ve tamoxifen-treated BCA. The latest study conducted by Barlett et al. was the first one involving node +ve patients. Bartlett et al. established the efficacy of this test in a study derived from the TEAM trial on node +ve and node -ve patients irrespective of nodal status treated with hormonal therapy consisting of either tamoxifen or exemestane (135).

Last, ASCO also is against the use of both the IHC4 and the Mammostrat in both node +ve or node -ve disease due to the fact that it has not been shown to be reproducible and their testing has been limited to few studies.

## Physicians’ Approach to Genomic Assays

**Figure 1:**
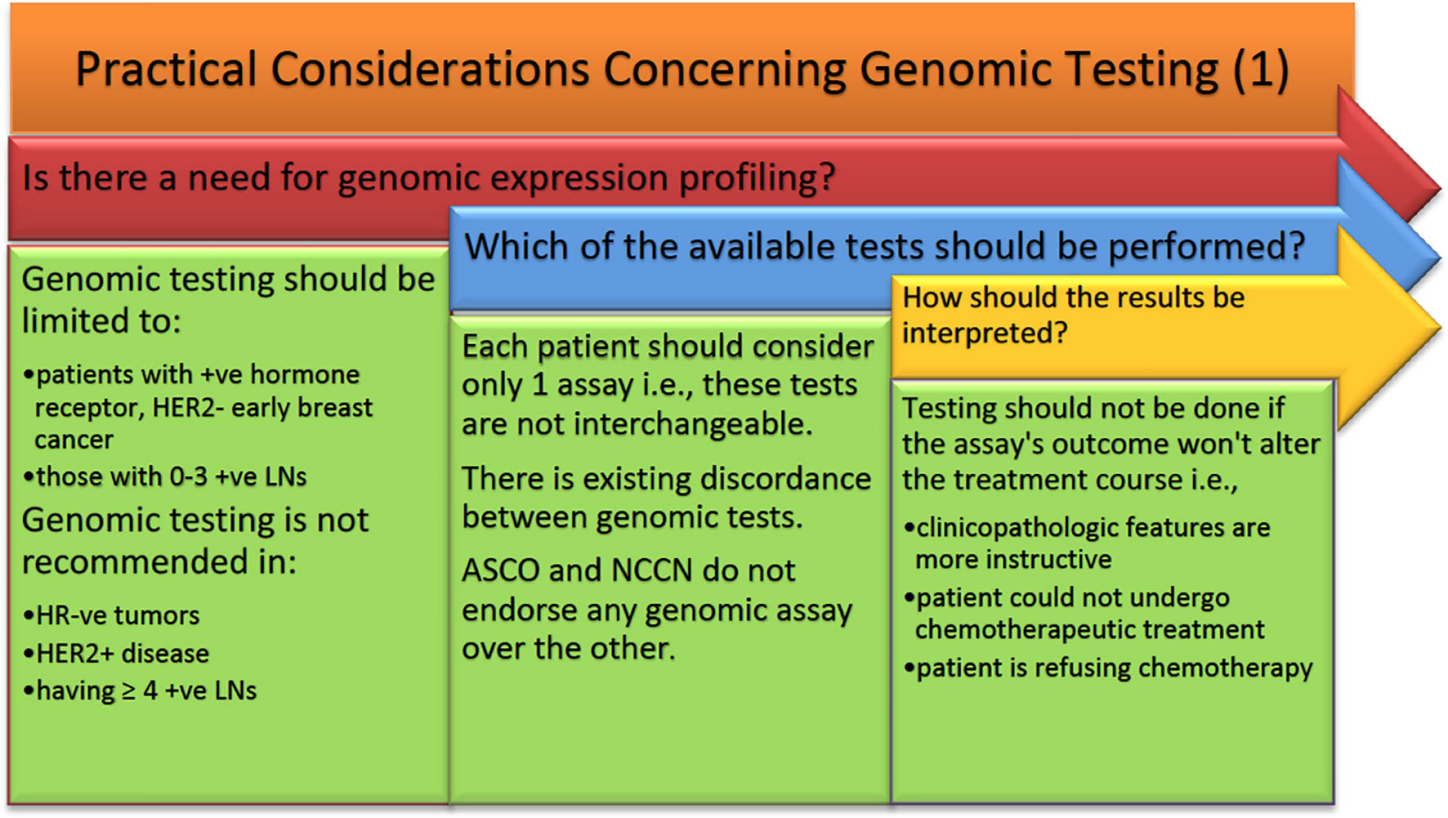
ASCO, American Society of Clinical Oncology; HER2, human epidermal growth factor receptor 2; NCCN, National Comprehensive Cancer Network.

Many studies have observed the difference in treatment course based on physician’s recommendations. Before we discuss the impact of the use of multiple genomic assays in node +ve early BCA, it is worth noting that not all patients are suitable for genomic testing. Some general principles should be taken into consideration when deciding who is the population of patients most likely to benefit from genomic testing (**Figure 1**). These principles are based on the BCTEG’s latest recommendations. Several reports in the literature deal with physician’s attitude towards multigene testing and how it alters the treatment plan. Indeed, a 1.7- and 1.5-fold increase in the application of Oncotype DX was observed among providers and patients respectively (136). This was observed when oncologists having a median number of connections of 4 were treating around 25,000 BCA patients. Here we present the most pertinent findings. Even though the BCTEG do not recommend the use of more than one genomic assay, Tsai et al. showed that almost 79% of physicians described greater confidence in their treatment plan when MammaPrint is applied on patients identified as having an intermediate RS by Oncotype DX (68). In that study, there was only a 5.8% reduction in physician’s confidence concerning their treatment recommendations after receiving MammaPrint results; the rest of the cases (N = 131) reported no effect on confidence after receiving the MammaPrint result (68).

First, It was concluded that not enough testing is being done for young females which could benefit from genomic assays (137). Poorvu et al. underwent a prospective study over 10 years studying early BCA in young patients (≤ 40 years). In this study, 32% of 575 stage I-III ER +ve/Her2 -ve BCA underwent Oncotype DX RS (137). Females aged less than 30 years old (OR = 0.49, p = 0.03) were less likely to undergo genomic assay testing compared to females aged 36–40 years old (OR = 0.87, p = 0.57) (137). Similarly, patients having tumors >2 cm (OR = 0.54, p = 0.007) or having a high-grade tumor (OR = 0.37, p < 0.0001) also utilized genomic assays less frequently. Last, 10% of patients having node +ve disease underwent genomic testing (OR = 0.14, P < 0.0001). Only 24% vs 57% vs 100% who had RS performed underwent CT representing patients with low, intermediate, and high RS, respectively (137).

Second, the decision to escalate or de-escalate treatment depends on many factors, and sometimes spares patients the financial burden and unintended side effects of CT (138). Assi et al. underwent a retrospective study including over 2 years showcasing the sway of genomic tests on adjuvant therapy decisions in early BCA patients (N = 75) (138). The three multigene assays utilized were Oncotype DX (50/75), Prosigna (11/75), and Endopredict (14/75). Genomic assays were helpful in two aspects. First, around 21 patients, who their physicians’ plans were initially equivocal, had their treatment plan decided after genomic test results were obtained (138). Second, out of the 26 patients who had their treatment plan changed, de-escalation of therapy took place in 25.33% of patients vs escalation of therapy took place in only seven patients (138). Some colleagues have even discussed the possibility of reducing the dose of CT. Leung and colleagues evaluated a total of 146 ER +ve LN -ve BCA patients who received genomic expression profiling along with recommendations before and after testing (139). Change in treatment recommendations along with reduction in intensity occurred 23.3 and 4.8%, respectively (139). There was a net relative reduction of 27.6% from 52.1 to 37.7% in chemotherapeutic recommendations; the net absolute reduction was 14.4% (P < 0.001) (139). Similar to the percentage of the change in recommendations, almost 30% of physicians felt that genomic expression profiling altered their management plan. Concerning the financial burden on patients, Waintraub and colleagues studied 227 female patients: out of which nearly 70% underwent genomic expression profiling. Unsurprisingly, chemotherapeutic treatment was administered less frequently in the gnomically profiled cohorts (19 vs 29%; P = 0.08) (140). Thus, a lower financial cost is observed when genomic expression profiling is applied in patients with stage II or grade 2/3 disease (140).

## Conclusion

BCA mortality has been reduced over the past years thanks to improved prognostic and predictive information aiding in decision-making. The use of genomic assays according to the latest guidelines has many added benefits to our management of patients. These benefits encompass personalized treatment, improving patients’ adherence, decreasing nervousness and conflicts associated with management decisions. Another benefit is the increased rate of cancer detection, which most of the times has been accurate. In some instances, however, these advances in BCA have resulted in false diagnoses. This has raised concerns regarding overdiagnosis, which eventually may result in overtreatment. Overtreatment is obviously problematic for several reasons. First, the side effects of CT are heavily worth bearing in mind when making a decision whether or not to initiate treatment. Self-reported side effects ranging from hot flushes, breathlessness, and weight gain to aching muscles, along with a variety of systemic toxicities with cardiac side effects such as dilated cardiomyopathy are major concerns when considering CT (141). Initiating treatment also had psychological repercussions on patients as well as cognitive decline in a subset of patients. The most commonly affected domains in these patients were verbal and visual memory, language, spatial capability, and executive functioning (142). Tailoring treatment is essential for many other reasons as well. Patients undergoing CT, particularly those receiving alkylating agents and anthracyclines, are at an increased risk of developing acute myeloid leukaemia. This was further enhanced by the use of radiotherapy (143). These patients were more likely to be hospitalized and necessitate additional costs. This brings to light the economic cost as well which at times acts as a barrier to treatment. In order to minimize overdiagnosis and its ramifications, we strongly encourage physicians to endorse these guidelines and adopt them in their own practice. While the authors agree that the morbidity accounted with CT becomes less of a burden when a patient’s life is saved, it is imperative that guidelines are not trespassed, and genomic assays are not overused as well. Ongoing studies are revealing the use of these assays in node +ve disease, and that is why it is recommended to be conservative with these assays while awaiting further results. In that sense, it is very important to check the results of the RxPonder trial, which is currently in phase 3. This trial will bring further insight into RS cut-offs at which a CT benefit can be detected in patients with node +ve disease. Another one is OPTIMA trial, also in phase 3, which will contribute to the care of patients with an ROR around 25. Two tables (**Tables 1** and **2**) briefly compare the multiple genomic assays.

**Table 1:** qRT-PCR, quantitative Reverse Transcriptase Polymerase Chain Reaction; CE, Conformité Européenne.

	*Oncotype DX*	*MammaPrint*		*Prosigna*	*EndoPredict*	*Breast Cancer Index*	*Genomic Grade Index*	*Immuno-histochemistry*	*Mammo-strat*
*Technique*	qRT-PCR (16 genes)	DNA micro-arrays (70 genes)	Nanostring nCoutner (50 genes)		qRT-PCR (8 genes)	qRT-PCR (7 genes)	DNA micro-arrays (97 genes) or qRT-PCR (4 genes)	IHC (4 IHC markers)	IHC (5 IHC markers)
*FDA Approved*	NO	YES	YES		NO	NO	NO	NO	NO
*CE-marked*	NO	YES	YES		YES	NO	NO	NO	NO
*Tissue Requirements*	formalin-fixed, paraffin-embedded tissue	formalin-fixed, paraffin-embedded tissue or frozen tissue	formalin-fixed, paraffin-embedded tissue		formalin-fixed, paraffin-embedded tissue	formalin-fixed, paraffin-embedded tissue	formalin-fixed, paraffin-embedded tissue	formalin-fixed, paraffin-embedded tissue	formalin-fixed, paraffin-embedded tissue
*Output Score*	Risk score and category (high vs. intermediate vs. low risk)	risk category (high vs. low risk)	Intrinsic subtype, risk of recurrence score (continuous)		Risk score and category (high vs. low risk)	Risk score and category (high vs. low risk)	Risk category (high vs. low risk)	Risk score and category (high vs. low risk	Risk score and category (high vs. low risk)

**Table 2:** ASCO, American Society of Clinical Oncology; EGTM, European Group on Tumor Markers; ER+, Estrogen-receptor-positive; ESMO, European Society for Medical Oncology; NCCN, National Comprehensive Cancer Network; DBCG, Danish Breast Cancer Group; CCTG, Canadian Cancer Trials Group.

	*Oncotype DX (21-gene assay)*	*MammaPrint(70-gene assay)*	*Prosigna(50-gene assay)*	*EndoPredict (12-gene assay)*	*Breast Cancer Index(7-gene assay)*
*Studies Used for Development*	NSABP-14 NSABP B-20 (5)	Netherlands Cancer Institute Cohort (144), RASTER study (52)	British Columbia Breast Cancer cohort (95)	GEICAM trial (105), ABCSG-6 (107), ABCSG-8 (98)	ATAC trial (96), Stockholm (111)
*Retrospective Studies*	NSABP B-20 (5), SWOG-8814 (26), TransATAC (22), SEER 18 (145), WSG-ADAPT (38)	Pooled database of 7 prospective trials (144)	ATAC (96), ABCSG-8 (98), DBCG (100)	ABCSG-6 (107), ABCSG-8 (98)	Stockholm (111), TransATAC (22), CCTG, MA.17 (112), aTTOM trial (120)
*Prospective Studies*	TAILORx, RxPONDER.	MINDACT, PROMIS	OPTIMA	NA	NA
*Prognostic/Predictive*	Yes for both, 10-year RR and adjuvant chemotherapy benefit	Only Prognostic, 10-year RR	Yes for both, 10-year RR and late recurrence (> 5 years) for patients HR+ve and LN-ve disease	Only prognostic, 10-year RR	Yes for both, 10-year RR and late recurrence (> 5 years) extended adjuvant endocrine therapy benefit
*Recommendations*	NCCN, ASCO, ESMO, St. Gallen, AJCC, NICE, EGTM	NCCN*, ASCO, ESMO, St. Gallen, EGTM	NCCN*, ASCO, ESMO, St. Gallen, EGTM	ASCO, ESMO, St. Gallen, EGTM	ASCO, St. Gallen, EGTM

NCCN* discussed but not specifically recommended.

## Concluding Recommendations

When it comes to the use of gene expression assays and BCA treatment recommendations, a few differences exist in the consideration of adding adjuvant systemic CT to adjuvant ET. According to ASCO, the MammaPrint assay, which has a level 1A clinical evidence, “may be used in patients with one to three +ve nodes and at high CR per MINDACT categorization to inform decisions on withholding adjuvant systemic CT due to its ability to identify a good prognosis population with potentially limited CT benefit” (42, 62). “However, such patients should be informed that a benefit of CT cannot be excluded, particularly in patients with greater than one involved lymph node” (42, 62). Recommendations for the use of biomarkers in treatment decision-making, which are split into first and second-generation signatures, differ when it comes to ESMO. The latest guidelines consider MammaPrint and Oncotype DX, both first-generation signatures, as prognostic for ER +ve, Her2 -ve tumors. Neoadjuvant CT is indicated if there is a high risk or score. Second-generation signatures such as Prosigna and Endopredict are prognostic when evaluating ER +ve, Her2 -ve tumors along with tumor size and nodal status. These signatures can be done on biopsies or surgical samples, and only then, neoadjuvant CT is indicated if there is a high risk or score. The following are the latest NCCN guidelines for different genomic assays when it comes to node +ve diseases. First, NCCN considers all gene expression assays in node +ve disease as prognostic, but it is yet unknown if these assays are predictive in patients with 1–3 +ve LNs. All gene expression assays have a level IIA NCCN category of evidence and consensus except for Mammaprint, which has an evidence level of category I.

## Author Contributions

MB and MI drafted the manuscript. CN, RH, MI, and MB contributed to the discussion section. HA conceived the idea for the paper. All authors contributed to the article and approved the submitted version.

## Conflict of Interest

The authors declare that the research was conducted in the absence of any commercial or financial relationships that could be construed as a potential conflict of interest.
