# Corrigendum: Genomic Assays in Node Positive Breast Cancer Patients: A Review

**DOI:** 10.3389/fonc.2021.686355

**Published:** 2021-04-14

**Authors:** Maroun Bou Zerdan, Maryam Ibrahim, Clara El Nakib, Rayan Hajjar, Hazem I. Assi

**Affiliations:** ^1^ Department of Internal Medicine, Naef K. Basile Cancer Institute, American University of Beirut Medical Center, Beirut, Lebanon; ^2^ Division of Internal Medicine, Massachusetts General Hospital and Harvard Medical School, Boston, MA, United States

**Keywords:** breast cancer, oncotype Dx, MammaPrint, PAM50, endopredict, breast cancer index, genomic grade index, immunohistochemistry

In the original article, there was an error. **MINDACT** trial is misspelled as **MINDCAT** on three occasions.

A correction has been made to ***Discussion, MammaPrint and BluePrint, MammaPrint, Role in Node Positive Disease***, paragraph 2:

“Here it is important to mention that panellists from BCTEG compared suggestions from the Early Breast Cancer Trialist’s Collaborative Group (EBCTCG) to the suggestions that a 10-year follow-up for the MINDACT is needed.”

A correction has been made to ***Discussion, MammaPrint and BluePrint, BluePrint, Validation Studies***, paragraph 4.

“In a study done by Viale et al. using the same patient population enrolled in the previously mentioned MINDACT trial, IHC/FISH was compared to both Amsterdam 80-gene and Amsterdam 70-gene (83).”

In the original article, there was a mistake in **Table 2** as published. The MINDACT trial is misspelled as MINDCAT. The corrected **Table 2** appears below:

**Table 2:** ASCO, American Society of Clinical Oncology; EGTM, European Group on Tumor Markers; ER+, Estrogen-receptor-positive; ESMO, European Society for Medical Oncology; NCCN, National Comprehensive Cancer Network; DBCG, Danish Breast Cancer Group; CCTG, Canadian Cancer Trials Group.

	*Oncotype DX (21-gene assay)*	*MammaPrint(70-gene assay)*	*Prosigna(50-gene assay)*	*EndoPredict (12-gene assay)*	*Breast Cancer Index(7-gene assay)*
*Studies Used for Development*	NSABP-14 NSABP B-20 (5)	Netherlands Cancer Institute Cohort (144), RASTER study (52)	British Columbia Breast Cancer cohort (95)	GEICAM trial (105), ABCSG-6 (107), ABCSG-8 (98)	ATAC trial (96), Stockholm (111)
*Retrospective Studies*	NSABP B-20 (5) SWOG-8814 (26), (TransATAC (22), SEER 18 (145), WSG-ADAPT (38)	Pooled database of 7 prospective trials (144)	ATAC (96), ABCSG-8 (98), DBCG (100)	ABCSG-6 (107), ABCSG-8 (98)	Stockholm (111), TransATAC (22), CCTG, MA.17 (112), aTTOM trial (120)
*Prospective Studies*	TAILORx, RxPONDER.	MINDACT, PROMIS	OPTIMA	NA	NA
*Prognostic/Predictive*	Yes for both, 10-year RR and adjuvant chemotherapy benefit	Only Prognostic, 10-year RR	Yes for both, 10-year RR and late recurrence (>5 years) for patients HR+ve and LN-ve disease	Only prognostic, 10-year RR	Yes for both, 10-year RR and late recurrence (>5 years) extended adjuvant endocrine therapy benefit
*Recommendations*	NCCN, ASCO, ESMO, St. Gallen, AJCC, NICE, EGTM	NCCN*, ASCO, ESMO, St. Gallen, EGTM	NCCN*, ASCO, ESMO, St. Gallen, EGTM	ASCO, ESMO, St. Gallen, EGTM	ASCO, St. Gallen, EGTM

NCNN* discussed but not specifically recommended.

In the original there was another error. The following are the latest NCCN guidelines for different genomic assays when it comes to node +ve diseases. First, NCCN considers all gene expression assays in node +ve disease as prognostic, but it is yet unknown if these assays are predictive in patients with 1–3 +ve LNs. All gene expression assays have a level IIA NCCN category of evidence and consensus except for PAM 50, which has an evidence level of category I. PAM50 should be MammaPrint. MammaPrint is the only test with level I evidence for LN+ patients. The correction has been made to **Concluding Recommendations:**

“All gene expression assays have a level IIA NCCN category of evidence and consensus except for Mammaprint, which has an evidence level of category I.”

In the original article the author’s citation for Clara El Nakib was incorrect. The correct citation is “El Nakib C”.

The authors apologize for these errors and state that these do not change the scientific conclusions of the article in any way. The original article has been updated.

